# An unbeatable opponent? Coaches’ perspectives on the impact of climate change in outdoor sports

**DOI:** 10.1186/s13102-025-01135-0

**Published:** 2025-04-22

**Authors:** Sven Schneider, Janina Reinmuth, Sophie Leer

**Affiliations:** Heidelberg University, Medical Faculty Mannheim, Center for Preventive Medicine and Digital Health (CPD), Division of Public Health, Social and Preventive Medicine, Röntgenstraße 7, D-68167 Mannheim, Germany

**Keywords:** Climate change, Athletes, Germany

## Abstract

**Background:**

Athletes in outdoor sports particularly experience several consequences of climate change.

**Objectives:**

To take up the experiences and expectations of coaches in outdoor sports regarding climate-related health risks in sport and to systematize them.

**Methods:**

This nationwide, cross-sectional study was conducted among adult outdoor sports coaches from the ten largest outdoor sports associations in Germany. Their experiences with climate-related changes where were collected and qualitative content analysis was conducted.

**Results:**

Out of 1,771 participating coaches, the content-analytical evaluation resulted in eight disjointed topics. These comprise heat-related risks, accident and injury risks, UV-related risks, respiratory risks, infection risks, mental risks and also positive effects of climate change. Besides, statements of respondents not expecting any significant changes due to climate change were recorded.

**Conclusions:**

In the course of climate change, sport-specific risks will continue to increase and especially the risk setting of outdoor sports will be confronted to them. In order to be able to practice sports successfully and safely in the future, the study results emphasize the necessity to develop comprehensive, flexible and cost-effective prevention concepts for climate adaptation in sports.

**Clinical trial number:**

Clinical trial number: The study protocol was pre-registered with the German Clinical Trials Registry (registration number DRKS00027815) on January 18, 2022 (https://drks.de/search/de/trial/DRKS00027815).

**Supplementary Information:**

The online version contains supplementary material available at 10.1186/s13102-025-01135-0.

## Introduction

Climate change especially endangers children, people with health impairments, multimorbid senior citizens, and those dependent on the outdoors [1–3]. Unlike other at-risk populations, outdoor athletes cannot escape worsening climate conditions by retreating indoors (e.g., into air-conditioned environments). A soccer match, track and field meet or cycling race cannot simply be transferred indoors. In fact, participating in these sports requires facing increasingly hazardous environments head-on.

Coaches occupy a key position in both the attainment of sporting success and the maintenance of the health of athletes. Outdoor sports coaches have divers experience dealing with the consequences of climate change [3]. In order to enable future generations to enjoy sport and promote their health, it is necessary to use this experience to develop comprehensive and sustainable intervention and prevention strategies.

This qualitative study aims to gather insights from a large collective of outdoor sports coaches regarding their experiences with and expectations of climate-related health risks in sports and systematize them. This research surpasses prior narrative approaches [4–6] by systemically capturing significant, experience- and practice-based insights.

## Methods

### Qualitative approach

Given the relatively new nature of the phenomenon, a qualitative approach was chosen to fully capture the wide range of potential effects. Specifically, this study utilizes qualitative content analysis as outlined by Kuckartz [7]. The structure of the method section follows the Standards for Reporting Qualitative Research (SRQR).

### Researcher characteristics and context

The study design and subsequent content analysis were led and supervised by the first author (SvS), a professor of social epidemiology with methodological training and over 25 years of experience in content analysis. His research focuses on the intersection of climate change and sports. All authors bring practical experience as outdoor athletes (SvS in track and field; JR in cycling, canoeing and kayaking; SL in cycling and alpine skiing). In addition to his academic role, SvS serves as a coaches’ instructor, while JR works as an outdoor sports coach. SvS trained co-author JR (B.A. in Sociology) and Janine Wolber (B.Sc. in Applied Health Sciences; see acknowledgements) in qualitative content analysis. The three authors worked collaboratively on the dataset at the same research center, holding regular meetings to review progress and reach consensus. The third author, SL (Medical doctor; B.Sc. in Business Informatics), was also responsible for participant recruitment and helped develop the coding framework.

### Sampling strategy

Participants were recruited through convenience sampling from both national and state-level general sports associations, as well as from organizations specific to the ten most popular outdoor sports in Germany. Outdoor sports were defined as those in which the competitions typically occur outdoors [8]. Using membership statistics from the German Olympic Sports Confederation [Deutscher Olympischer Sportbund] (DOSB) for 2023, the ten largest outdoor sports associations were identified [9]. These included the German Soccer Association [Deutscher Fußball-Bund] (DFB), German Tennis Association [Deutscher Tennis Bund] (DTB), German Alpine Association [Deutscher Alpenverein] (DAV), German Track and Field Association [Deutscher Leichtathletik-Verband] (DLV), German Equestrian Federation [Deutsche Reiterliche Vereinigung] (FN), German Golf Association [Deutscher Golf Verband] (DGV), German Lifeguards Association [Deutsche Lebens-Rettungs-Gesellschaft] (DLRG), German Ski Association [Deutscher Skiverband] (DSV), German Sailing Association [Deutscher Segler-Verband] (DSV) and German Cyclists’ Federation [Bund Deutscher Radfahrer] (BDR). In order to reach as many coaches as possible, a call for participation in the study was distributed via websites, digital newsletters and association notices (e.g., printed or online magazines) of the associations. The call for participation included general information about the study and a web link to complete a Computer Aided Web Interview (CAWI). Publicly available email and phone directories were also used to directly contact coaches by author SL. The participants who were recruited according to this procedure were also provided with the same information, which included a link to the CAWI.

### Ethical considerations

This study was conducted in accordance with the Declaration of Helsinki. Ethics approval was obtained from the ethics committee of the Medical Faculty Mannheim, Heidelberg University (AZ 2021 − 653, November 16, 2021). Additionally, the study protocol was pre-registered with the German Clinical Trials Registry (registration number DRKS00027815) on January 18, 2022 (https://drks.de/search/de/trial/DRKS00027815).

### Data collection methods

Data was gathered through a standardized, asynchronous CAWI using Lime-Survey software. In the introductory section of the survey, the first and last authors (SvS, SL) introduced themselves as research assistants from Heidelberg University and clearly stated the scientific purpose of the study. Nationwide data collection was conducted from May 1, 2022, to June 30, 2023, with the database locked at the conclusion of the collection period. The subsequent qualitative data analysis was finalized on February 6, 2024.

### Data collection instruments and technologies

At the outset of the survey, participants were provided with basic information about the study and were asked to give informed consent. They were then prompted to answer the key open-ended question: “What health effects do you think climate change will have on outdoor sports enthusiasts in the next 10 years?” This specific time frame was chosen to focus on a period relevant for developing prevention measures. Respondents were offered a large, unrestricted text field to enter their free-text answers and were encouraged to address multiple points to capture a wide range of opinions.

In addition to the open-ended question, coach-specific data (such as gender, age, sport, experience and qualifications) and club-specific data (such as training frequency, group age, group size and club size) was collected through closed-ended questions. Before launching the survey, the CAWI was reviewed through expert consultations (*n* = 4, including a sports sociologist, a physician for physical and rehabilitative medicine, a coach and a state sports association manager) and piloted with a standard pre-test (*n* = 8 coaches, who were excluded from the final sample). Feedback from these expert reviews and the pre-test was incorporated into the final version of the CAWI. A translated version of the relevant parts of the questionnaire can be found as supplementary file (see below). In addition, the original questionnaire will be made available by the corresponding author upon request.

### Region and study units

The study was conducted in Germany, which has a temperate climate with average monthly temperatures ranging between − 1 °C and + 17 °C. The country features diverse topographical regions, including coastal areas along the North and the Baltic seas and alpine areas, among others. Eligible participants were coaches working in competitive or recreational sports within Germany’s ten most popular outdoor sports. Participants were required to be at least 18 years old and fluent in German.

### Data processing

After the data collection phase, all responses were copied verbatim into separate text files. The files were then imported into MAXQDA (version 12.3, VERBI Software GmbH, Berlin) for analysis.

### Data analysis

The analysis process began with SvS und JR reviewing the extensive text material to identify potential overarching themes. JR subsequently performed an inductive and systematic coding of all text passages, identifying further main and corresponding subthemes. To standardize the coding process, a coding guide was developed, detailing coding rules to ensure consistent assignment of responses to distinct categories. So-called “anchor examples” were provided to clarify procedures. When ambiguous free-text statements arose, SvS and JR discussed these until a consensus was reached, and the examples were incorporated into the coding guide. JR then applied this coding scheme to the remainder of the text data. A medical doctor (SL) reviewed the final scheme.

For quality assurance, the themes, subthemes and selected statements were translated into English by a native speaker, and translated back into German by the authors to ensure accuracy. In the results section, quotes from coaches are generally presented verbatim, with only spelling errors corrected for readability. In cases where this was necessary for data protection reasons, statements were paraphrased. Each quote is accompanied by information on the participant’s gender (male or female), age, license level (ranging from pre-qualification to A-license), target group (amateur or competitive) and type of sport. In addition to the qualitative analyses, descriptive quantitative analyses were conducted to characterize the coach cohort. These quantitative analyses were performed using IBM SPSS Statistics (version 29.0.0.0, IBM Corp., Armonk, USA).

### Techniques to enhance trustworthiness

To ensure the trustworthiness of the research, a second coder (JW) independently coded the complete free-text responses of 100 randomly selected participants. This coding was compared with the work of the first coder (JR), resulting in an inter-rater reliability of 0.75 (kappa coefficient). Several additional measures were implemented to ensure the quality of data collection, including CAPTCHA protection, real-time IP and plausibility checks and the use of digital data to eliminate input errors.

The analysis process adhered to the quality criteria outlined by Ahmed [10] to ensure credibility, transferability, dependability and confirmability. Credibility was enhanced by triangulating our results with quantitative studies in the discussion section. Transferability and dependability were ensured through detailed documentation and transparency of the methodology, as demonstrated by pre-registration and thorough reporting in this publication. Confirmability was supported by the authors’ practical experience as outdoor athletes and coaches.

## Findings

The sample included 1,771 coaches with an average age of 44.18 ± 14.48 years, comprising 66.2% male, 33.6% female and 0.2% non-binary respondents. On average, coaches had 15.33 ± 11.57 years of coaching experience, with 86.1% holding an accredited coaching license (C-license or higher). Most coaches led training sessions at least once a week (81.6%). The average team size was 10.13 ± 9.23 and 26.8% were made up of minors. The membership of the sports clubs varied, with 43.9% having 300 or fewer members, 34.5% having 301-1,000 members and 21.7% having over 1,000 members. The respondents represented various sports, including soccer (*n* = 187), tennis (*n* = 363), mountain sports (*n* = 137), track and field (*n* = 210), horseback riding (*n* = 202), golf (*n* = 113), swimming (*n* = 203), skiing (*n* = 114), sailing (*n* = 111), cycling (*n* = 100) and other outdoor sports (*n* = 31).

**Fig. 1:**
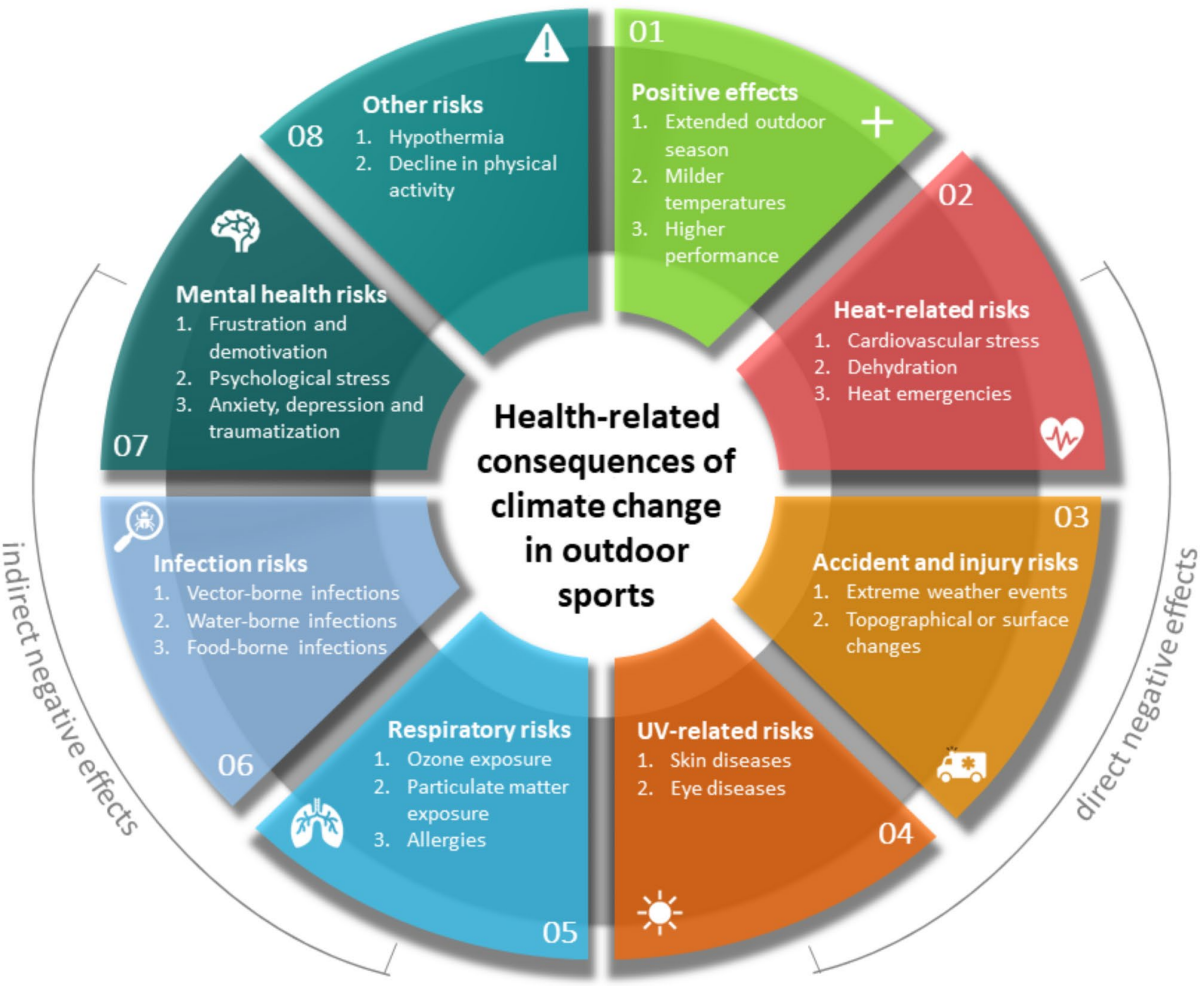
Health-related consequences of climate change resulting from content analysis evaluation

The content analysis of the coaches’ responses regarding the health impacts of climate change on outdoor athletes over the next ten years revealed eight distinct themes, which are outlined in Fig. 1 (Fig. 1). The data, comprising a total text volume of 17,505 words, was initially divided into positive and negative effects, with the negative impacts significantly outweighing the positive. The negative effects were then further divided into main categories and subcategories, as depicted in Fig. 1 (Fig. 1).

### Positive effects

A small number of respondents highlighted positive aspects of climate change. These included the potential for an extended outdoor sports season due to warmer temperatures:

“*Actual warming would have advantages in terms of extending the season.*” (ID1162, not specified, sailing).

Endurance athletes, in particular, may benefit from fewer instances of extremely cold outdoor conditions:

“*Positive for cyclists: milder temperatures enable continuous training at high level even in winter.*” (ID240, f, 46, C-lic. comp., cycling).

In addition, some technical sports, such as those involving explosive movements, could benefit from improved muscle contraction and a reduced risk of injury in warmer temperatures:

“*For many athletes in technical disciplines (jumping*,* throwing)*,* higher temperatures could even have advantages (lower risk of muscle injuries [*and of*] cooling down during competition breaks).*” (ID379, f, 34, C-lic. comp., track and field).

### Negative effects

#### Heat-related risks

The most frequently cited negative effect was heat. Coaches noted that the rising temperatures and more frequent heatwaves associated with climate change would increase the risk of heat-related health issues. Heat stress on the cardiovascular system, particularly during endurance exercise, was a major concern:

“*Older […] athletes will experience high cardiovascular stress more quickly.*” (ID453, m, 40, B-lic. comp., track and field).

Dehydration was another common issue, with typical symptoms such as fatigue, drowsiness and confusion becoming prevalent:

“*Heat has a major impact on golfing in particular - simply because of the age structure of our sport. Older people have greater problems in the heat. […] Combined with too little water intake*,* we’ve had the ambulance on the golf course several times due to dehydration.*” (ID2413, m, 45, B-lic. amat., golf).

Finally, the risk of heat-related emergencies, such as sunstroke, heat exhaustion and exertional heat stroke, also increases for athletes and coaches:

“*The main issue is the coaches*,* who are sometimes so busy with others that they no longer pay attention to themselves. We’ve had instructors missing during summer break due to sunstroke.*” (ID341, not specified, sailing).

#### Accident and injury risks

Coaches also anticipated an increase in accident and injury risks due to extreme weather events. Heavy rain, storms and falling branches pose immediate threats for most outdoor athletes, while mountain and ski sports are at greater risk of mudslides, rockfalls and avalanches. In water sports, dangerous fluctuations in water levels and flow velocities were predicted:

“*Injuries caused by weather phenomena: During the tornado in Kiel in 2021*,* several sailors were on the water and several rowers [were] injured.*” (ID498, f, 29, C-lic. amat., sailing).

In addition to these acute events, extreme weather conditions also altered risk factors in both the medium and long term. Interviewees highlighted an increased risk of injury from dried-out grass fields and uneven surfaces caused by drought, as well as erosion-related changes in topography, leading to unpredictable challenges in mountain and climbing routes. Prolonged periods of low water levels could result in misjudgment of water depths, further increasing the potential for accidents:

“*[Due to climate change*,* ] rocks sometimes become so brittle that existing bolts fly out of the rock and you either set your own belays or don’t get on the route at all.*” (ID500, m, 45, pre.-lic. amat., mountain sports).

#### UV-related risks

The increase in UV exposure driven by climate change is linked to both immediate and long-term health risks in sport. Coaches frequently cited sunburn (UV erythema, dermatitis solaris) and sun allergies as acute health risks. They also emphasized the chronic dangers associated with increased UV exposure, particularly the heightened risk of developing various forms of skin cancer. Additionally, there is an expected rise in UV-related eye conditions, such as cataract, due to increased sun exposure.

“*Higher UV exposure causes more severe sunburn and increases the risk of skin cancer.*” (ID 1368, f, 59, C-lic. amat., soccer).

#### Respiratory risks

The respiratory risks mentioned by the coaches surveyed can be categorized into three key areas. The first involves inflammatory processes and respiratory diseases triggered by increased ozone exposure in the air.

“*Higher ozone exposure leads to irritation of the mucous membranes*,* […] coughing*,* fatigue*,* loss of energy and level of performance.*” (ID 1368, f, 59, C-lic. amat., soccer).

Coaches frequently mentioned the dangers of ground-level ozone, with fewer references to particulate matter, although both were recognized as significant threats:

“*Rising particulate matter and ozone levels may [cause] long-term damage to respiratory organs.*” (ID385, m, 57, C-lic. amat., track and field).

The third category concerns atopic diseases. Changes in the ecosystem are expected to increase pollen count, concentration and allergenicity. Combined with high temperatures and air pollution, these factors heighten the risk of exacerbating allergic conditions, such as rhino conjunctivitis or allergic asthma, particularly for athletes.

“*Respiratory diseases like bronchial asthma will increase due to air pollution and very warm*,* dry air.*” (ID396, m, 59, A-lic. comp., cycling).

Athletes, especially those engaged in endurance sports, are particularly vulnerable due to their increased respiratory minute volume during intense physical activity.

#### Infection risks

The surveyed coaches expressed concerns about an increase in infection risks for athletes, primarily due to climate change. The first risk they highlighted involved vector-borne diseases, with warmed conditions leading to the proliferation of vectors such as ticks and mosquitoes. Diseases like tick-borne encephalitis (TBE), Lyme disease and leishmaniasis were specifically mentioned as potential threats:

“*Warmer winters lead to more parasites in summer. Ticks (borreliosis) continue to spread.*” (ID1701, not specified, horseback riding).

Secondly, coaches feared that rising bacterial loads in bodies of water could elevate infection risks for athletes involved in water sports. Pathogens such as leptospires, along with the increased presence of cyanobacteria in warmer waters, were noted as growing concerns:

“*Less water in lakes and higher water temperature lead to bacteria in the water*,* which can be harmful to health.*” (ID1634, not specified, Swimming).

Lastly, the risk of contamination and the presence of harmful germs in the athletes’ drinking water and provisions, especially during periods of high temperatures, was also a significant concern.

#### Mental health risks

The coaches identified three key areas where climate change could have mental health impacts on athletes. Firstly, deteriorating environmental conditions, such as heatwaves and other extreme weather events, were seen as potential causes for frustration and a decline in motivation. The inability to plan training sessions or competitions due to cancellations or postponements could further demotivate athletes.

“*Lack of motivation for an athletic challenge.*” (ID2104, m, 62, C-lic. amat., soccer).

Secondly, the direct impact of heat itself was noted to increase stress levels—not only for athletes but also for sports animals:

“*Increased stress level and thus different behavior of the outdoor sport horse […] and thus also increased risk of accidents.*” (ID1166, f, 29, B-lic. comp., horseback riding).

Lastly, climate-related extreme events such as avalanches, floods, storms and mudslides were identified as triggers for anxiety, psychological stress and even trauma. These events, even when causing mild injuries, could have lasting mental health consequences:

“*Injuries or*,* in extreme cases*,* fatalities [could] also cause psychological stress*,* anxiety*,* trauma and depression.*” (ID2168, not specified, golf).

#### Other risks

Additional risks identified by the coaches included hypothermia and frostbite, particularly in mountain and winter sports, due to unexpectedly severe temperature fluctuations. Moreover, the anticipated changes in climate could make both summer and winter sports increasingly difficult to practice, leading to reduced participation in these activities. This decline in physical activity could, in turn, contribute to the rise of lifestyle-related diseases such as obesity, diabetes and hypertension.

“*Societally*,* increasing climate changes could lead to a reduction in the occurrence of sports*,* so that less exercise would lead to an increase in so-called civilization diseases such as diabetes*,* back problems*,* obesity*,* high blood pressure […] and many others*,* not only in adults but also [in] children and especially in less physically active people.*” (ID435, m, 52, C-lic. comp., track and field).

Table 1 provides an overview of the structure of the main and subcategories of the effects discussed (Table 1). While quantitative evaluations are uncommon in qualitative studies, at least the number of text passages forming each main category is included for reference (Table 1).

Neither Fig. 1 nor Table 1 includes statements from respondents who did not anticipate significant changes as a result of climate change—whether due to skepticism about climate change itself or the belief that athletes would adapt over time:

“*Climate change is currently only taking place on an ideological basis.*” (ID679, not specified, tennis).

Additionally, a number of respondents admitted feeling underinformed or overwhelmed by the topic:

“*No idea*,* I haven’t engaged with the subject enough.*” (ID867, m, 48, B-lic. comp., swimming).

## Discussion

### Summary of key findings

This study presents climate-related health risks in sports from the perspective of those directly impacted, drawing on what is likely the largest study collective to date: Surveying over 1,700 coaches from a variety of outdoor sports in Germany reveals a broad spectrum of opinions on the health risks posed by climate change. Outdoor sports will face six primary risk areas: heat-related risks (e.g., dehydration), accident and injury risks (e.g., avalanches), UV-related risks (e.g., skin cancer), respiratory risks (e.g., allergens), infection risks (e.g., ticks) and mental health risks (e.g., trauma following accidents). Some coaches also acknowledged potential positive effects, particularly the prospect of milder weather extending seasons. However, others did not foresee any major changes, either due to skepticism about climate change or a belief that athletes will adapt over time.

### Positioning the results in national and international research

The health consequences of climate change for populations globally, nationally and regionally have become increasingly differentiated and systematized in recent years [2, 11, 12]. Naturally, many of these consequences also impact athletes, including direct effects like heat exposure and indirect effects like air pollution and vector- and water-borne diseases [13] (Goldblatt, 2022). However, several serious health impacts associated with climate change—such as mortality risks, malnutrition and supply crises caused by droughts, migration, social conflict and economic loss of productivity—do not significantly affect sports [2, 12, 14].

In contrast, this study identifies sport-specific risks that are less relevant to the general population and, consequently, have received limited attention in previous research. Examples include exertional heat strokes, ophthalmological risks, drowning, accidents from lightning strikes and falling branches, unpredictable hazards in mountainous regions due to shifting route difficulties, injury risks on dried-out playing surfaces, leishmaniasis infections, water contamination in water sports, psychological trauma following sports accidents, frostbite from alpine weather changes and declining physical activity levels [15, 16]. These topics, rarely discussed or only briefly mentioned in existing literature, demonstrate the need for a sport-specific approach to climate change risks.

This study serves as an initial step in that direction. The next step should involve deeper, sport-specific risk analyses (for activities like soccer, cycling, water sports, winter sports, etc.).

### Weaknesses and strengths of the study

As is typical of qualitative research, this study cannot be considered representative of outdoor sports as a whole. Certain outdoor sports that are less popular in Germany, such as American football and baseball, were not included. However, the broad range of sports covered, the consistency in training and competition conditions across disciplines and the large number of study participants help to mitigate these limitations. Another constraint is the study’s geographic focus, which is limited to Germany. While Germany’s varied landscape—including coastal regions along the North and Baltic Seas, lowlands, numerous lakes and alpine areas—offers diverse climate conditions, the impacts of climate change in regions outside the mid-latitudes may differ significantly.

The key strength, and a unique feature of this study, is the first systematic recording of climate-related health risks in sports through in-depth interviews with those directly affected, rather than relying on a purely narrative review. This publication thus integrates the firsthand knowledge and experience of over 1,700 coaches, offering a valuable perspective on this evolving issue.

### Outlook

As climate change progresses, sport-specific risks will inevitably rise, particularly in outdoor sports. To ensure that we can continue to enjoy these activities safely and successfully in the future, it is critical to develop comprehensive, flexible and affordable prevention strategies—whether in stadiums, on sports fields, in the mountains or on and in the water. Fortunately, there are already well-developed and successfully tested models for climate adaptation in sports [17–19]. These models recommend a combination of structural measures (e.g., installing sun sails, setting up sunshades during breaks, chemically treating nearby water sources), organizational measures (e.g., adjusting training schedules to cooler times of the day, revising competition rules, modifying clothing policies, introducing additional cooling breaks, redesigning endurance competitions as nighttime events, etc.) and personalized measures (e.g., providing certified jerseys and sportswear with UPF 50 + ratings, placing stickers in changing rooms reminding athletes to apply sunscreen before practice and check for ticks after practice, supplying cooling jackets, cold packs, water sprayers and sunscreen dispensers).

These models are relatively easy to tailor to specific sports, and their widespread dissemination and promotion are crucial. By doing so, sports associations and clubs worldwide can avoid starting from scratch, conserving both human and financial resources in the ongoing battle against the harmful health effects of climate change.

**Table 1:** Themes and subthemes with anchor examples and frequencies from content analysis

Main theme	Frequencies	Subtheme	Anchor examples
Positive effects	19	Extended outdoor season Milder temperatures Higher performance	Earlier start and later end to the outdoor season, longer outdoor training periods Positive effects during winter training, better breathing conditions, reduced physical strain due to cold Improved performance in technical disciplines, lower risk of muscle injuries due to higher temperatures
Heat-related risks	1,023	Cardiovascular stress Dehydration Heat emergencies	Increased cardiovascular stress, impaired blood circulation, symptoms like dizziness, malaise, unconsciousness Dehydration, complications like thrombosis, embolism, electrolyte imbalances, headaches, cramps, increased need for water Sunburn, sunstroke, heatstroke, heat exhaustion, overheating, collapse
Accident and injury risks	165	Extreme weather events Topographical or surface changes	Unpredictable weather (heavy rain, storms, thunderstorms), falling trees, landslides, avalanches, mudslides, rockfalls, drowning from circulatory problems or large temperature differences while swimming/diving, sailing-related water hazards Increased injury risk due to drought, hard outdoor surfaces
UV-related risks	669	Skin diseases Eye diseases	Dermatitis solaris, UV-induced inflammatory reactions, risk of skin cancer, epithelial (especially basal cell carcinoma and spinalioma) and non-epithelial (especially melanoma) skin tumors, carcinomas Eye diseases, irritation, risk of cataracts
Respiratory risks	350	Ozone exposure Particulate matter exposure Allergies	Respiratory diseases and breathing difficulties due to higher ozone levels Respiratory diseases like bronchial asthma increasing due to air pollution Pollen-induced allergies, asthma, hay fever
Infection risks	40	Vector-borne infections Water-borne infections Food-borne infections	Tick-borne encephalitis (TBE), diseases transmitted by arthropods (ticks, mosquitoes), leishmaniasis Increased risk from bacterial contamination of water, blue-green algae affecting water quality Contamination of drinking water and provisions
Mental health risks	49	Frustration and demotivation Psychological stress Anxiety, depression and traumatization	Lack of motivation, reduced drive to exercise, fewer opportunities to participate in sports Increased stress levels, inability to cope with stress, stress from anxiety Anxiety, depression, psychological trauma
Other risks	517	Hypothermia Decline in physical activity	Hypothermia, frostbite Reduced participation in sports, decreased physical performance, increased lifestyle diseases (such as obesity, diabetes and hypertension), difficulty maintaining winter sports due to lack of snow

## Electronic supplementary material

Below is the link to the electronic supplementary material.

Supplementary Material 1

## Data Availability

The original questionnaire and the dataset supporting this study are available in German on request from the corresponding author [SvS].
