# Supplementary material for: An unbeatable opponent? Coaches’ perspectives on the impact of climate change in outdoor sports
Source: BMC Sports Sci Med Rehabil. 2025 Apr 22;17:89. doi: 10.1186/s13102-025-01135-0 (PMC12016389; doi:10.1186/s13102-025-01135-0)
Supplement: Supplementary file 1 — Supplementary Material 1 [file 13102_2025_1135_MOESM1_ESM.pdf]

What are the aims of the study?

Climate change has a massive impact on the environment, society and health. This makes it all the more surprising that climate change has so far received comparatively little attention in sport and sports medicine. Yet not only competitive athletes, but also recreational athletes will be directly confronted with the health effects of climate change in the future. What's more, athletes are considered a risk group for the health consequences of climate change. Outdoor sports are particularly affected.

The aim of this independent scientific survey is to record risk perception, health literacy and practice in your club with regard to the central climate-related health risks of "heat extremes" and "UV exposure" and to derive appropriate recommendations for action for German popular sport. To this end, we surveyed you as a trainer in one of the 10 outdoor sports with the highest number of members in the German Olympic Sports Confederation (according to official membership statistics).

What requirements must be met to participate in the study?

You are at least 18 years old You are a coach/trainer in one of the following outdoor sports: soccer, tennis, alpine sports, athletics, horse riding, golf, DLRG, skiing, sailing, cycling Your place of residence is within Germany

How does the study work?

The aim of this written online survey is to ask trainers about their experiences and opinions regarding climate-related health risks and their prevention options.

Do I have a personal benefit?

Yes, the processed results and prevention recommendations will be made available to you at the end of the study as a report for your work as a trainer or for your sport.

What risks are associated with participation?

Completing the questionnaire takes about 25 minutes once and does not involve any risks for you.

We would like to point out that participating in the online survey via smartphone while actively participating in road traffic is prohibited by law and involves risks for yourself and other road users.

Voluntariness/resignation

Participation in the study is voluntary. If you wish to participate, we ask you to agree to the declaration of consent below. You can revoke this consent at any time in writing or verbally without giving reasons and without incurring any disadvantages. If you wish to withdraw your consent, please contact the study management. Please note that data that has already been included in scientific evaluations can no longer be deleted/destroyed at your request.

Will I incur any costs as a result of my participation? Will I receive payment or compensation for my expenses?

Participation in the study is free of charge for you. You will not receive any payment. As a reward for taking part, 2 x 1 Amazon voucher worth €50 each will be raffled off among all participants.

Further information

For further information, general results and the outcome of the study, please contact the study leader:

Prof. Dr. Sven Schneider

Center for Preventive Medicine and Digital Health Baden-Württemberg (CPD-BW)

Ludolf-Krehl-Str. 7-11, 68167 Mannheim

Phone: 0621/3839910

E-Mail: sven.schneider@medma.uni-heidelberg.de

We are grateful for your participation in this research project!

With kind regards

Sophie Leer

Doctoral candidate

Center for Preventive Medicine and Digital Health Baden-Württemberg (CPD-BW)

Medical Faculty Mannheim of the University of Heidelberg

Ludolf-Krehl-Str. 7-11, 68167 Mannheim

E-Mail: Leer@stud.uni-heidelberg.de

2 more important notes on processing the questionnaire:

It is known that LimeSurvey experiences a session timeout after some time. This means that the questionnaire has to be completed again and the results have not been saved if you take a break of around 25 minutes in between - i.e. if you have not entered another answer. So if you take a longer break when completing the questionnaire, remember to save your answers in good time (button top right, "Continue later"). Mandatory questions are marked with a red asterisk (*).

The survey begins with the general health effects of climate change for outdoor sports enthusiasts.

A1. **How likely do you think it is that climate change will have an impact on the health of outdoor sports enthusiasts within the next 10 years?**

Probability in %|0%|100% [ ]

A2. **What health effects do you think climate change will have on outdoor sports enthusiasts in the next 10 years?**

Please indicate several points in keywords.

[ ]

A3. **In which sport are you mainly active as a coach?**

- Mountain sports (hiking, climbing, mountaineering) [ ]
- Swimming [ ]
- Soccer [ ]
- Golf [ ]
- Athletics [ ]
- Cycling [ ]
- Equestrian sports [ ]
- Sailing [ ]
- Skiing [ ]
- Tennis [ ]
- - Others - [ ]

We would now like to learn more about the application of certain prevention measures in your training courses.

B1. **Please imagine a cloudless, sunny day for all of the following questions.**

How often do the following statements apply to training that you typically carry out outdoors with your training group under these conditions?

If you train several sports groups, please focus on the sports group you train most frequently.

If individual statements do not apply to your sport, please set the slider to 0 “is never realized”.

The training takes place in the shade. |is never realized|is always realized

The training breaks take place in the shade. |is never realized|is always realized

If the training would take place during lunchtime, I move it to the morning or evening hours. |is never realized|is always realized

If the training were to take place during midday, it would take place in the shade with medium and high UV irradiance (UV index 3-7). |is never realized|is always realized

If the training were to take place during lunchtime, I would cancel it if the UV irradiance was very high (UV index ≥8). |is never realized|is always realized

The majority of sportspeople use sun protection products. |is never realized|is always realized

The majority of athletes apply sunscreen at least 20-30 minutes before training. |is never realized|is always realized

The majority of athletes apply sunscreen evenly to all free areas of the skin. |is never realized|is always realized

The majority of sportspeople use a sunscreen with an adequate sun protection factor (at least SPF 30 for children and at least SPF 20 for adults). |is never realized|is always realized

The majority of athletes apply the sunscreen in a sufficiently thick layer (approx. 2mg/cm2). For an adult, for example, 1 heaped tablespoon of sunscreen is required for a quarter of the body. |is never realized|is always realized

The majority of athletes reapply sunscreen after 2 hours of training at the latest. |is never realized|is always realized

The majority of athletes use a waterproof sunscreen. |is never realized|is always realized

The majority of athletes use a sunscreen for the lips. |is never realized|is always realized

The majority of athletes wear sunglasses. |is never realized|is always realized

The majority of athletes wear headgear during training. |is never realized|is always realized

The majority of athletes wear headgear during training breaks. |is never realized|is always realized

The majority of athletes wear clothing that at least covers their shoulders. |is never realized|is always realized

The majority of athletes wear long pants. |is never realized|is always realized

Medical personnel are present at organized competitions. |is never realized|is always realized

LimeSurvey

Lastly, we would like to collect some information concerning your person and your coaching activities.

L1. Sex

- Male ☐
- Female ☐
- Other ☐

L2. Age

[| | | | | | | | |]

L3. Which is the highest training/coach license you possess?

- None ☐
- Pre-qualification (e.g. assistant coach, team leader or comparable) ☐
- Juleica (Jugendleiter-Card) ☐
- C-License – popular/mass sports ☐
- C-License – competitive sports ☐
- B-License – popular/mass sports ☐
- B-License – competitive sports ☐
- A-License – popular/mass sports ☐
- A-License – competitive sports ☐
- Diploma ☐

L4. In total, how many years have you been active as a coach of the aforementioned sport?

[| | | | | | | | |]

L5. During a typical main season, how often do you give training sessions in the aforementioned sport?

- Several times per day ☐
- Once per day ☐
- Several times per week ☐
- Once per week ☐
- Several times per month ☐
- Once per month ☐
- Less than the aforementioned ☐
- Currently not at all ☐

L6. Which audience do you mainly coach in the aforementioned sport?

- Children ☐
- Adolescents ☐
- Adults <60 years ☐
- Adults ≥ 60 years ☐

L7. On average, what size is your training group in the aforementioned sport?

[| | | | | | | | |]

LimeSurvey

L8. **In which state do you mainly work as a coach of the aforementioned sport?**

- Baden-Württemberg ☐
- Bavaria ☐
- Berlin ☐
- Brandenburg ☐
- Bremen ☐
- Hamburg ☐
- Hesse ☐
- Mecklenburg-Vorpommern ☐
- Lower Saxony ☐
- North Rhine-Westphalia ☐
- Rhineland-Palatinate ☐
- Saarland ☐
- Saxony ☐
- Saxony-Anhalt ☐
- Schleswig-Holstein ☐
- Thuringia ☐

L9. **What is the postal code of the club in which you primarily work as a coach of the aforementioned sport?**

L10. **How big is the club in which you primarily work as a coach of the aforementioned sport?**

- ≤ 100 members ☐
- 101-300 members ☐
- 301-1.000 members ☐
- 1.001-2.500 members ☐
- > 2.500 members ☐

Dear trainer/coach,

thank you for your participation in the online survey “Outdoor Sprts and Climate Change”. Your answers have been stored. You can close the browser window now.

Specific suggestions for training during heat can be found here: DGSP • Deutsche Gesellschaft für Sportmedizin und Prävention e.V.
For general information concerning the topic “Health and Climate Change”, we refer to the site of the Robert Koch Institute: RKI – Klimawandel und Gesundheit (German only)

For questions about the questionnaire or general questions about the study, feel free to contact me via email (Leer@stud.uni-heidelberg.de)

We are grateful for your participation in this research project!

With kind regards

Sophie Leer

Doctoral candidate

Center for Preventive Medicine and Digital Health Baden-Württemberg (CPD-BW)

Medical Faculty Mannheim of the University of Heidelberg

Ludolf-Krehl-Str. 7-11, 68167

Mannheim

E-Mail: Leer@stud.uni-

heidelberg.de
